# A strategic approach to managing Immune Effector Cell-Associated Hematotoxicity (ICAHT) during chimeric antigen receptor T-cell therapy

**DOI:** 10.1007/s44313-026-00143-4

**Published:** 2026-05-19

**Authors:** Sang Eun Yoon

**Affiliations:** Division of Hematology-Oncology, Department of Medicine, Samsung Medical Center, Sungkyunkwan University School of Medicine, 81 Irwon-Ro Gangnam-Gu, Seoul, 06351 Republic of Korea

To the Editor:

Although T-cell engager therapies, including bispecific antibodies and chimeric antigen receptor (CAR) T-cell therapy, have revolutionized treatment of hematological malignancies, immune effector cell-associated Hematotoxicity (ICAHT) remains a significant clinical challenge [1, 2]. Effective ICAHT management requires a proactive strategy beyond reactive monitoring that integrates initial bone marrow capacity with post-infusion hematological trends to categorize patient risk [3–5].

A critical first step is to use the CAR-HEMATOTOX score before lymphodepletion. While this model excels in identifying patients at high risk for prolonged neutropenia (Score ≥ 2), its clinical utility is primarily its high negative predictive value. Rather than serving as an absolute mandate for prophylactic intervention, a high CAR-HEMATOTOX score should be viewed as an indicator for intensified surveillance to ensure timely therapeutic intervention [3] (Table 1).

**Table 1:** Clinical framework for prediction and management of Immune Effector Cell Associated Hematotoxicity (ICAHT)

Classification	CAR-HEMATOTOX	N-ICAHT	T-ICAHT
**Primary aim**	Risk stratification and Predictive modeling	Grading of neutropenia	Grading of Thrombocytopenia
**Assessment timing**	Prior to lymphodepletion (base-line)	Post-CAR-T infusion (Early: 0—30 days, Late: ≥ 30 days)	Post-CAR-T infusion (Early: 0—30 days, Late: ≥ 30 days)
**Key variables**	ANC, Hb, PLT, ferritin, and CRP	ANC Depth and duration	PLT Depth and duration
**High-risk**	Score ≥ 2	Early ANC ≤ 500/uL, for ≥ 14 days ANC ≤ 100, for ≥ 7 days	Early, Late PLT < 20 × 10^9^/L
		Late ANC 100—500/uL	
**Clinical intervention**	Intensified surveillance for early intervention	Neutropenia-driven G-CSFsupport	Transfusion support and relapse monitoring
**Prognostic impact**	Predicts prolonged hematotoxicity	Short-acting G-CSF: based on clinical neutropenia status Long-acting G-CSF: only after confirming inflammatory safety	Independent predictor of inferior OS and relapse rate
**Key perspective**	A signal for vigilance, not an absolute mandate for prophylaxis	Faster recovery is not always better	A surrogate for bone marrow exhaustion and immune failure

*Abbreviation*: *ANC* Absolute Neutrophil Count, *CAR-T* Chimeric Antigen Receptor T-cell, *CRP* C-reactive Protein, *CRS* Cytokine Release Syndrome, *Hb* Hemoglobin, *ICAHT* Immune Effector Cell-Associated Hematotoxicity, *ICANS* Immune Effector Cell-Associated Neurotoxicity Syndrome, *N-ICAHT* Neutropenic ICAHT, *OS* Overall Survival, *PFS* Progression-Free Survival, *PLT* Platelet, *T-ICAHT* Thrombocytopenic ICAHT, *OS* overall survival

Management of neutropenia (N-ICAHT) underscores the principle that “faster recovery is not always better.” While short-acting granulocyte colony-stimulating factor (G-CSF) allows for tighter control of myeloid recovery, prophylactic use of long-acting G-CSF before CAR-T infusion has been shown to significantly increase the risk of Grade ≥ 2 cytokine release syndrome (CRS) (HR 2.15, *P* = 0.02) [6]. We thus advocate a personalized, neutropenia-driven approach. While short-acting G-CSF can be initiated in response to the patient's immediate hematologic status, long-acting G-CSF should be strictly deferred until inflammatory safety is ensured following resolution of active CRS/ICANS [4] (Table 1).

Our focus must expand to include thrombocytopenia (T-ICAHT) as a primary prognostic driver. In a core cohort of 744 patients with B-NHL, severe early T-ICAHT (Grade 3—4) was a powerful independent predictor of poor survival. Specifically, grade 3—4 T-ICAHT was associated with a 2-year overall survival (OS) of only 35%, compared with 67% in grade 0 (*p* < 0.001). This survival deficit is driven by a 60% relapse rate rather than by non-relapse mortality. We hypothesize that severe thrombocytopenia serves as a surrogate for bone marrow reservoir exhaustion, reflecting a compromised immune microenvironment that fails to maintain long-term immunosurveillance [5] (Table 1).

In conclusion, optimizing CAR-T cell therapy outcomes necessitates a three-fold strategy: forecasting risk via CAR-HEMATOTOX, avoiding premature G-CSF intervention in N-ICAHT, and prioritizing T-ICAHT monitoring as a sentinel for relapses and long-term prognosis. Successful implementation of this framework will require establishing a robust nationwide registry to systematically collect and analyze real-world ICAHT data. Such a national-scale platform will serve as an essential foundation to validate these risk-adapted approaches and represent a cornerstone of balanced and effective patient care in this era of CAR-T cell therapies.

## Data Availability

No datasets were generated or analysed during the current study.
